# Pre-Operative Delta-MELD is an Independent Predictor of Higher Mortality following Liver Transplantation

**DOI:** 10.1038/s41598-019-44814-y

**Published:** 2019-06-05

**Authors:** George Cholankeril, Andrew A. Li, Brittany B. Dennis, Chiranjeevi Gadiparthi, Donghee Kim, Alice E. Toll, Benedict J. Maliakkal, Sanjaya K. Satapathy, Satheesh Nair, Aijaz Ahmed

**Affiliations:** 1 Division of Gastroenterology and Hepatology, Stanford University School of Medicine, Stanford, CA USA; 2 Department of Internal Medicine, Stanford University School of Medicine, Stanford, CA USA; 3 Department of Medicine, Saint George’s Hospital, University of London, London, UK; 4 Division of Gastroenterology and Hepatology, University of Tennessee Health Science Center, Memphis, TN USA; 5 Department of Research, United Network for Organ Sharing, Richmond, VA USA

**Keywords:** Risk factors, Liver, Liver diseases

## Abstract

Clinical decompensation immediately prior to liver transplantation may affect post-liver transplant (LT) outcomes. Using the serial Model for End-Stage Liver Disease (MELD) scores recorded in the United Network for Organ Sharing national registry (2010–2017), we analyzed post-LT mortality among adult LT recipients based on the degree of fluctuation in MELD score during the 30-day period prior to LT surgery. Delta-MELD (D-MELD) was defined as recipient MELD score at LT minus lowest MELD score within the preceding 30 days. Impact of D-MELD as a continuous and categorical variable (D-MELD 0–4, 5–10, >10) on early, 30-day post-LT mortality was assessed. Overall, a total of 12,785 LT recipients were analyzed, of which 8,862 (67.9%) had a pre-operative D-MELD 0–4; 2,574 (20.1%) with a D-MELD 5–10; and 1,529 (12.0%) with a D-MELD > 10. One-point incremental increase in pre-operative D-MELD (adjusted HR, 1.07, 95% CI: 1.04–1.10) was associated with higher 30-day post-LT mortality. Moreover, pre-operative D-MELD > 10 was associated with nearly a two-fold increased risk for 30-day post-LT mortality (adjusted HR, 1.89, 95% CI: 1.30–2.77) compared to D-MELD 0–4. The increased risk of pre-LT mortality associated with severity of clinical decompensation assessed by the magnitude of pre-operative D-MELD persists in the early post-LT period.

## Introduction

In patients with liver failure in the setting of cirrhosis, acute-on-chronic liver failure (ACLF) is a clinical entity characterized by sudden onset and rapid progression of underlying hepatic dysfunction which may be accompanied by multi-system organ failure with high risk of short-term mortality (50–90%) without prompt supportive measures and liver transplant (LT) surgery^1–5^. ACLF often impacts the Model for End-Stage Liver Disease (MELD) score, a validated objective predictor for early pre-LT mortality and the primary allocation criteria for liver transplantation in the United States (US)^6–8^.

Although a ≥5 points rise in MELD score over 30 days in the setting of ACLF has been shown to predict pre-LT waitlist mortality^9,10^, the data are conflicting in terms of post-LT survival, in part due to widely varying time frames that have been evaluated^9,11–15^. Using the Organ Procurement Transplant Network/United Network for Organ Sharing (OPTN/UNOS) registry, Northup *et al*. in 2004 reported that a preoperative rise in MELD score of ≥5 within 30 days of liver transplantation was not predictive of post-LT survival^14^. Another study analyzing 69,643 waitlist registrants found that a rise in MELD score of >30% during a seven-day period was associated with an increase in waitlist mortality but lacked association with post-LT mortality^16^. In a single center analysis, Gyori *et al*. demonstrated that patients with a rise in MELD score of >10 over their time on the waitlist tended to experience worse 1-, 3-, and 5-year post-LT survival^11^, which was later replicated in a large Eurotransplant registry analysis^12^. However, the relationship between sudden changes in MELD score immediately prior to LT surgery and outcomes was not examined.

Utilizing the serial laboratory MELD scores from the OPTN/UNOS database, we aim to assess in a standardized manner the impact of sudden rise in recipient MELD score prior to LT surgery on early post-transplant mortality. We studied Delta-MELD (D-MELD) defined as recipient MELD score at the time of LT surgery minus lowest MELD score within the preceding 30 days.

## Methods and Materials

### Data source

Our study used data from OPTN/UNOS registry, which includes national data on all organ donations, waitlist registrants and LT recipients in the US. All patient and transplant center identifiers were excluded. This study was exempt from Institutional Review Board (Stanford University Research Compliance Office) review at our institution, as the data set used in the analysis was completely de-identified.

### Patients

Using serial MELD scores reported within the OPTN/UNOS registry, we analyzed post-LT mortality among adult (age ≥ 18) LT recipients from January 1, 2010 to December 31, 2017 who experienced a sudden pre-operative rise in their MELD score prior to LT surgery. We utilized an objective parameter, the D-MELD which is the absolute difference in recipient MELD score at the time of LT surgery and lowest MELD score (MELD_i_) tested and reported during the 30 days prior to LT surgery. All LT recipients had a calculated or laboratory MELD score ≥ 15 at the time of LT surgery. LT recipients without a reported MELD_i_ within 30 days preceding LT were excluded. In addition, LT recipients with concomitant hepatocellular carcinoma, allocation MELD exception score higher than their calculated MELD score at LT, Status 1A, living donor liver transplantation and simultaneous multiple organ transplantation were excluded.

### Definitions

MELD scores were calculated utilizing serial MELD score parameters including serum sodium (mmol/L), serum creatinine (mg/dL), serum bilirubin (mg/dL), and international normalized ratio for prothrombin time, which were provided by OPTN/UNOS. The standard UNOS formula which incorporated sodium as of January 1, 2016^17^ was used to determine serial MELD scores^18^.

$$\begin{array}{rcl}MEL{D}_{(i)} & = & {0.957}\times Log(creatinine\,mg/dL)\\  &  & +\,{0.378}\times Log(bilirubin\,mg/dL)\\  &  & +\,{1.120}\times Log+{0.643}\\ MELD & = & MEL{D}_{(i)}+{1.32}\,\ast \,(137-Na)\\  &  & \mbox{--}\,[{0.033}\,\ast \,MEL{D}_{(i)}\,\ast \,(137-Na)]\end{array}$$

The minimum serum creatinine value was set to 1.0 mg/dL and the maximum serum creatinine value was set to 4.0 mg/dL. Serum creatinine value of 4.0 mg/dL was assigned to subjects if they underwent two or more dialysis treatments or received 24 hours of continuous veno-venous hemodialysis within 7-days prior to serum creatinine test. Data on the date of initiation of dialysis or continuous veno-venous hemodialysis were unavailable. The minimum serum sodium value was set to 125 mmol/L and the maximum serum sodium value was set to 137 mmol/L.

### Variables

Clinical characteristics at time of LT surgery included demographic data (age, gender, race/ethnicity), etiology of liver disease, ascites, hepatic encephalopathy (HE), renal failure on dialysis, change in creatinine (mg/dL), change in bilirubin (mg/dL), change in INR, initial MELD at the time of waitlist addition and MELD score at LT were included. Donor characteristics included liver donor risk index^19^ and utilization of donation after cardiac death (DCD) donors.

### Outcome

The primary outcome was to evaluate the impact of sudden rise in the magnitude of pre-operative D-MELD on early post-LT mortality. Mortality was assessed at 7-day, 30-day and 1-year following liver transplantation. D-MELD was analyzed as both a categorical (D-MELD 0–4, D-MELD 5–10, D-MELD > 10) and continuous variable (1-point increment).

### Statistical analysis

Cox proportional hazards regression models were fitted for each variable separately to determine association with post-LT mortality as the dependent variable. Separate models were constructed to assess post-LT mortality at 7-day, 30-day and 1-year intervals. In these models, patients were censored on the date of last follow-up if death had not occurred, and all observations were censored at 7-day, 30-day or 1-year interval depending on the time-specific model. Forward stepwise regression was performed with entry and exit criteria set to *P* = 0.10. Variables with biological plausibility for association with survival were included even if *P* value was above the *P* value threshold. Demographic and clinical characteristics were compared using Chi-square test for categorical variables and Kruskal-Wallis for quantitative variables. Cumulative incidence and 95% confidence intervals (CI) for post-LT mortality estimates were calculated while accounting for competing risk of graft failure. Log-rank was used to test differences between multiple cohorts. In a pre-planned sensitivity analysis, we analyzed recipients’ MELD scores capped and uncapped above 40. Statistical significance was met with a *P* value < 0.05. All statistical analyses were performed using the Stata 14 statistical package (StataCorp, College Station, TX).

## Results

### Clinical characteristics

**Figure 1:**
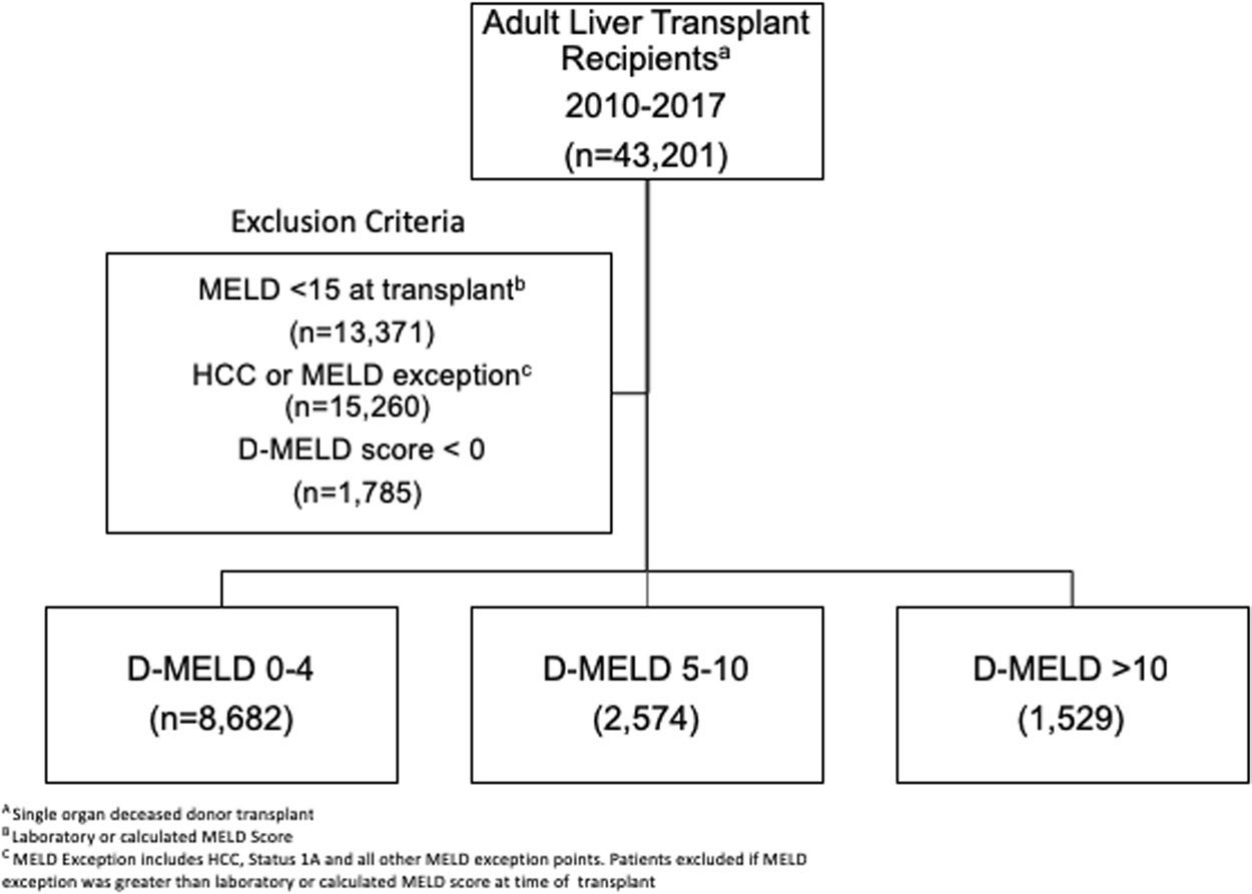
Study identification, inclusion, and exclusion criteria utilized in constructing D-MELD cohorts.

From January 1, 2010 to December 31, 2017, there were 12,785 adult LT recipients analyzed that met the inclusion criteria as shown in Fig. 1. Demographics and clinical characteristics of the total cohort are demonstrated in Table 1. The majority of patients were Caucasian males with a median age of 55 (IQR, 48–61). Chronic hepatitis C virus (HCV)-related cirrhosis and alcoholic cirrhosis constituted nearly half of all LT recipients and more than one-quarter of recipients were on renal dialysis at the time of LT surgery. Less than 5% of patients received a DCD liver graft. Recipients had a median MELD score at LT of 31 (IQR, 24–39) and median D-MELD of 2 (IQR, 0–6). Separate analyses were performed with recipients’ MELD scores capped at 40 and uncapped above 40. No statistical differences in outcomes were observed between the analyses. Therefore, the results reported utilize recipients’ capped MELD scores in accordance with the current UNOS allocation scoring system. Of the 12,785 recipients included in this study, 8,862 (67.9%) recipients had a pre-operative D-MELD 0–4, 2,574 (20.1) recipients had a D-MELD 5–10, and 1,529 recipients had a D-MELD > 10 within 30 days prior to LT surgery. Recipients with a D-MELD > 10 had a higher percentage of Hispanics, HCV-related cirrhosis, renal dialysis dependency, severe HE or ascites at the time of LT surgery (Table 1). Recipients with a D-MELD > 10 utilized a lower proportion of DCD donors. No statistical difference (*P* > 0.05) was seen in median age, gender or median LDRI score among the three cohorts. Differences in mean change in MELD score parameters including sodium, creatinine, bilirubin, and INR are tabulated in Table 2.

**Table 1:** Clinical characteristics of liver transplant recipients categorized by D-MELD.

	Total Cohort (n = 12,785)	D-MELD 0–4 (n = 8,862)	D-MELD 5–10 (n = 2,574)	D-MELD > 10 (n = 1,529)	*P* value
**Median Age (IQR**)	55 (48–61)	55 (48–61)	56 (49–62)	57(50–62)	0.1528
**Male, (%)**	8239 (64.4)	5726 (64.6)	1591 (61.8)	922(60.3)	0.3372
**Ethnicity, (%)**					
Caucasian	9058 (70.8)	6180 (71.2)	1830 (71.1)	1048 (68.5)	0.0029
Black	1363 (10.7)	1002 (11.5)	246 (9.6)	115 (7.5%)	
Hispanic	1833 (14.3)	1153 (13.3)	386 (15.0)	294 (19.2)	
Asian	352 (2.8)	230 (2.6)	74 (2.9)	48 (3.1)	
**Etiology of Liver Disease, (%)**					
ALD	2885 (22.6)	2109 (24.3)	520 (20.2)	256 (16.7)	0.0002
HCV	3076 (24.1)	1994 (22.5)	632 (24.6)	450 (29.4)	
ALD/HCV	652 (5.1)	442 (5.1)	121 (4.7)	89 (5.8)	
NASH	1941 (15.2)	1342 (15.5)	397 (15.4)	202 (13.2)	
Cholestatic Liver Disease	1342 (10.5)	851 (9.8)	292 (11.3)	199 (13.0)	
Other	2889 (22.6)	1944 (22.4)	612 (23.8)	333 (21.8)	
**Median Liver Donor Risk Index (IQR)**	1.36 (1.12, 1.65)	1.36 (1.12, 1.65)	1.35 (1.12, 1.62)	1.34 (1.13, 1.63)	0.8350
**Donation after Cardiac Death, (%)**	588 (4.6)	442 (5.3)	104 (4.1)	42 (2.8)	0.0258
**Renal Dialysis, (%)**	3652 (28.6)	2300 (26.5)	639 (24.8)	713 (46.6)	<0.0001
**Ascites, (%)**	11151 (87.2)	7489 (86.3)	2263 (87.9)	1399 (91.5)	0.0004
**Severe Hepatic Encephalopathy, (%)**	2052 (16.1)	1234 (14.2)	455 (17.7)	363 (23.7)	<0.0001

ALD, alcoholic liver disease, D-MELD, delta change in Model for End-Stage Liver Disease score; HCV, chronic hepatitis C virus infection; INR, international normalized ratio for prothrombin time; IQR, interquartile range; NASH, nonalcoholic steatohepatitis.

**Table 2:** Study cohort MELD scores and differences in MELD score parameters.

	Total Cohort	D-MELD 0–4 (n = 8,862)	D-MELD 5–10 (n = 2,574)	D-MELD > 10 (n = 1,529)	*P* value
Median Lowest MELD (IQR)	25 (21–32)	26 (21–36)	26 (22–30)	23 (20–26)	<0.0001
Median MELD score at Transplant (IQR)	31 (24–39)	27 (23–37)	33 (29–39)	40 (36–40)	<0.0001
Median D-MELD (IQR)	2 (0–6)	1 (0–2)	7 (5–8)	14 (12–17)	<0.0001
Mean Change in Creatinine (SD)	0.23 (1.06)	0.01 (0.85)	0.49 (1.04)	1.18 (1.51)	<0.0001
Mean Change in Sodium (SD)	0.40 (5.0)	0.09 (3.81)	0.52 (6.26)	1.95 (7.50)	<0.0001
Mean Change in Bilirubin (SD)	2.83 (7.00)	0.97 (3.93)	4.21 (7.12)	11.23 (12.05)	<0.0001
Mean Change in INR (SD)	0.42 (1.80)	0.14 (1.19)	0.74 (2.35)	1.42 (2.86)	<0.0001

D-MELD, delta change in Model for End-Stage Liver Disease score; INR, international normalized ratio for prothrombin time; IQR, interquartile range; MELD, Model for End-Stage Liver Disease score; SD, standard deviation.

### Mortality

**Figure 2:**
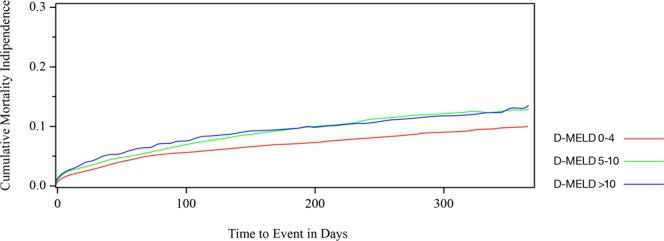
Cumulative incidence post-transplant mortality curves in cohorts with pre-operative D-MELD < 5 (0–4), D-MELD 5–10 and D-MELD > 10 taking the competing risk of graft failure and re-transplantation into account.

Post-LT cumulative mortality incidence rates at 7-day, 30-day and 1-year interval among recipients are depicted in the Supplementary Table. Recipients with a D-MELD 5–10 had significantly higher mortality rates than those with a lower D-MELD between 0–4 (*P* < 0.05). In addition, both LT recipients with a D-MELD 5–10 and a D-MELD > 10 demonstrated 1-year mortality rates exceeding 10%. Differences in post-LT mortality rates between these three cohorts are further illustrated in Fig. 2 with >10 D-MELD LT recipients demonstrating the highest post-LT mortality rates. Separate univariate and multivariate survival models analyzed post-LT mortality at 7-day, 30-day and 1-year. Univariate analyses demonstrated that D-MELD as a continuous variable with 1-point increments was associated with higher mortality within 1-year of LT surgery (Table 3). Multivariate analyses were adjusted for recipient age, gender, ethnicity, etiology of liver disease, LDRI, DCD donor, renal dialysis, ascites, severe HE, MELD score at the time of LT, mean change in MELD score parameters and OPTN/UNOS Region. After adjusting for these covariates, D-MELD as a continuous variable was still associated with higher post-LT mortality in each survival model at 7-day, 30-day and 1-year. Using similar survival models, D-MELD was analyzed as a categorical variable on post-LT mortality. After adjusting for covariates including MELD score at the time of LT surgery, recipients with a D-MELD 5–10 and a D-MELD > 10 had a significantly higher 30-day and 1-year post-LT mortality (Table 3). Table 4 demonstrates significant predictive risk for 30-day post-LT mortality. At 30-day assessment, D-MELD > 10 was the strongest predictor for post-LT mortality (D-MELD 0–4, reference; D-MELD 5–10, HR, 1.34 95% CI: 1.03–1.76; D-MELD > 10, HR 1.89, 95% CI: 1.30–2.77; *P* = 0.001). Other significant predictors for 30-day post-LT mortality included utilization of DCD donors (HR, 1.67; 95% CI: 1.09–2.54, *P* = 0.018), increasing LDRI (HR 1.40, 95% CI: 1.21–1.60, *P* < 0.001), and renal dialysis (HR, 1.37, 95% CI: 1.08–1.75, *P* = 0.010). Graft failure was analyzed as a post-LT outcome separate from mortality, however no association was noted between higher D-MELD and acute graft failure within 1-year of liver transplant surgery.

**Table 3:** Univariate and multivariate Cox regression models analyzing D-MELD as a continuous and categorical variable on early post-transplant mortality.

	Univariate HR (95% CI)	*P* value	Multivariate HR^a^ (95% CI)	*P* value
**D-MELD** ^**b**^				
7-Day	1.03 (1.01–1.05)	0.0122	1.07 (1.02–1.12)	0.002
30-Day	1.04 (1.02–1.06)	<0.001	1.07 (1.04–1.10)	<0.001
1-Year	1.03 (1.02–1.04)	<0.001	1.04 (1.02–1.05)	<0.001
**7-Day**				
D-MELD 0–4	Reference		Reference	
D-MELD 5–10	1.38 (1.02–1.88)	0.0388	1.40 (0.99–2.00)	0.0600
D-MELD > 10	1.43 (1.05–2.07)	0.0484	1.82 (1.09–3.06)	0.0266
**30-Day**				
D-MELD 0–4	Reference		Reference	
D-MELD 5–10	1.38 (1.10–1.66)	<0.001	1.34 (1.03–1.76)	0.0316
D-MELD > 10	1.92 (1.45–2.39)	<0.001	1.89 (1.30–2.77)	0.0010
**1-Year**				
D-MELD 0–4	Reference		Reference	
D-MELD 5–10	1.32 (1.16–1.51)	<0.001	1.31 (1.13–1.52)	<0.001
D-MELD > 10	1.40 (1.20–1.64)	<0.001	1.33 (1.06–1.67)	0.0131

CI, confidence interval D-MELD, delta change in Model for End-Stage Liver Disease Score; HR, hazard ratio.

^a^Multivariate analysis adjusted for recipient age, gender, ethnicity, etiology of liver disease, liver donor risk index, donation after cardiac death, renal dialysis, ascites, hepatic encephalopathy, MELD score at transplant, mean change in MELD score parameters (sodium, creatinine, bilirubin, INR) and UNOS Region.

^b^D-MELD analyzed as a continuous variable with one-point increments.

**Table 4:** Multivariate Cox regression analyses to predict risk factors for 30-day post-transplant mortality.

	HR^a^ (95% CI)	*P* value
**Recipient Age**	1.03 (1.02–1.04)	<0.0001
**Male**	1.09 (0.87–1.35)	0.4589
**Ethnicity**		
Caucasian	Reference	
African-American	0.91 (0.63–1.31)	0.6058
Hispanic	1.03 (0.76–1.40)	0.8337
Asian	1.28 (0.75–2.19)	0.3669
**Etiology of Liver Disease**		
ALD	Reference	
HCV	0.97 (0.71–1.33)	0.8575
NASH	1.05 (0.74–1.48)	0.7924
Cholestatic Liver Disease	1.16 (0.68–1.97)	0.5876
**Donation after Cardiac Death**	1.67 (1.09–2.54)	0.0180
**Liver Donor Risk Index**	1.40 (1.21–1.60)	<0.0001
**Renal dialysis**	1.37 (1.08–1.75)	0.0099
**Ascites**	1.19 (0.81–1.74)	0.3712
**Severe Hepatic Encephalopathy**	1.16 (0.84–1.61)	0.3667
**DMELD**		
D-MELD < 5 (0–4)	Reference	
D-MELD 5–10	1.34 (1.03–1.76)	0.0316
D-MELD > 10	1.89 (1.30–2.77)	0.0010

ALD, alcoholic liver disease; CI, confidence interval D-MELD, delta change in Model for End-Stage Liver Disease Score; HCV, chronic hepatitis C virus infection; HR, hazard ratio; NASH, nonalcoholic steatohepatitis.

^a^Additionally adjusted for recipient MELD score at transplant, change in MELD score parameters (sodium, creatinine, bilirubin, international normalized ratio) and UNOS Region.

## Discussion

In this large national cohort, we demonstrate a sudden rise in pre-operative D-MELD is associated with a significantly lower early post-LT survival. Sudden increase in the MELD score ranging between 5–10 to as high as >10 were associated with a significantly higher 30-day and 1-year post-LT mortality after adjusting for confounding factors, including absolute MELD score at transplant. These observations may play an important role in the organ allocation decision-making process to optimize the post-LT outcomes.

While the primary finding of an association between sudden changes in MELD and lower post-LT survival has been hypothesized in the transplant community, it has to our knowledge not previously been shown in a large cohort study. In addition, this primary finding is somewhat in contrast to previously published work in this area. In particular, while Northup *et al*. in 2004 reported that pre-operative rise in D-MELD was associated with early post-LT mortality, this association was not present after adjusting for absolute MELD. However, the study was limited by the fact that it was conducted in the pre-MELD and pre-sodium-MELD eras. Our analysis also excluded patients with HCC, acute liver failure including those listed as Status 1A and a MELD < 15 at time of LT surgery, allowing for an accurate assessment for ACLF. In two more recent analyses, Gyori *et al*. did not specifically tackle this question, but instead focused on changes in MELD over the entire waitlist period rather than a sudden change (*e.g*., preceding 30 days) in MELD preceding LT surgery^11,12^ and did not adjust for several known risk factors known to adversely impact LT survival such as absolute MELD at transplant, LDRI, and potential complications of decompensated cirrhosis (HE, severe ascites, hemodialysis).

The data presented here demonstrate a robust association between sudden changes in D-MELD within 30 days of liver transplantation and short-term post-LT mortality beyond that which is explained solely by the absolute MELD. Higher D-MELD, reflecting a sudden ACLF within the 30 days preceding LT surgery, and increased post- LT mortality may be directly related through several possible mechanisms. ACLF may result from either the primary hepatic decompensation from worsening liver disease or as a manifestation of an underlying disease process such as severe infection or multi-organ failure. The poor outcomes in LT recipients associated with a sudden and significant rise in pre-LT D-MELD versus relatively low D-MELD may reflect the consequences of ongoing underlying pathology that continues to progress post-operatively without affecting absolute pre-LT MELD score. Infectious complications are a major cause of early post-LT morbidity and mortality^20^. Known pre-operative risk factors for infection following liver transplantation include worsening renal function, long-term hemodialysis, and rising bilirubin levels, which are all captured by high D-MELD^21,22^. In addition, LT recipients hospitalized in the intensive care unit requiring vasopressor or ventilator support immediately prior to LT surgery are at a higher risk for post-LT mortality within one-year^23,24^. Therefore, developing management strategies focused on identifying risk factors pre-emptively and preventing ACLF will be key areas for improvement. More importantly, the same strategies can help improve organ allocation and resource utilization by identifying high-risk candidates prior to liver transplantation^25^. However, the apparent increased mortality associated with increased D-MELD over that of the absolute pre-transplant MELD would need to be weighed against a concurrent possible increase in waitlist mortality to better risk-stratify patients who are acutely decompensating. In our analysis, donor quality as measured by donation after cardiac death and LDRI score were the only controllable variables affecting early post-transplant mortality and should be heavily considered in this patient population.

Our study was limited by its retrospective design. In collaboration with OPTN/UNOS, we developed a customized dataset and were able to evaluate serial MELD scores reported by transplant centers to update MELD scores of waitlisted patients. However, any dynamic changes in the MELD scores of waitlisted patients that were not reported by liver transplant programs were not evaluated in this study. Therefore, it is plausible that a decline in pre-LT MELD score and D-MELD in a subset of LT recipients was not captured and we therefore did not evaluate the impact of a decline in pre-operative MELD score or negative D-MELD on early post-transplant mortality. In addition, we did not analyze the potential risk factors associated with higher pre-operative D-MELD because we were unable to quantitatively assess the variables known to impact ACLF such as infection, cardiovascular deterioration, vasopressor support, and portal hypertension complications including variceal bleeding, spontaneous bacterial peritonitis, hepatorenal syndrome, and hepatopulmonary syndrome. Other perioperative variables were also not captured in the UNOS/OPTN dataset, such as infection, surgical complication, pulmonary or cardiovascular compromise. In addition, we were unable to determine pharmacologic therapy including antiviral therapy and associated virologic response to therapy. Furthermore, the cause of death in LT recipients was not analyzed due to a significant percentage of missing data (>30%). All prior studies using the OPTN/UNOS database studied only recipient’s MELD score at the time of registration and transplant. However, we studied serial MELD scores in LT recipients utilizing a customized OPTN/UNOS dataset.

Using a national transplant registry, we demonstrate that a higher pre-operative D-MELD is associated with significantly increased risk of early post-LT mortality. Moreover, LT recipients with a pre-operative D-MELD > 10 were noted to have a two-fold increase in 30-day post-LT mortality, even after adjusting for MELD score at transplant. We suggest that an increased risk of pre-LT mortality associated with ACLF may not be captured by absolute MELD at the time of LT surgery. Therefore, D-MELD can identify waitlist patients awaiting liver transplantation who are at increased of early post-LT mortality. To this end, further studies are needed to confirm our findings and utilize these findings in the assessment of and allocation of organs for prospective LT recipients.

## Supplementary information

Cumulative incidence post-transplant mortality rates among D-MELD cohorts.

## Data Availability

The datasets generated during and/or analyzed during the current study are available from the corresponding author on reasonable request.
