# Supplementary material for: Pre-Operative Delta-MELD is an Independent Predictor of Higher Mortality following Liver Transplantation
Source: Sci Rep. 2019 Jun 5;9:8312. doi: 10.1038/s41598-019-44814-y (PMC6549161; doi:10.1038/s41598-019-44814-y)
Supplement: Supplementary file 1 — Cumulative incidence post-transplant mortality rates among D-MELD cohorts. [file 41598_2019_44814_MOESM1_ESM.docx]

**Title:**

**Pre-Operative Delta-MELD is an Independent Predictor of Higher Mortality following Liver Transplantation**

**Authors:**

George Cholankeril MD^1^, Andrew A. Li MD^2^, Brittany B. Dennis, PhD^3^, Chiranjeevi Gadiparthi MD^4^, Donghee Kim MD^1^, Alice E. Toll MS^5^, Benedict J. Maliakkal MD^4^, Sanjaya K. Satapathy MD^4^, Satheesh Nair MD^4^, Aijaz Ahmed MD^1^

**Author Affiliations:**

^1^ Division of Gastroenterology and Hepatology, Stanford University School of Medicine, Stanford, CA

^2^ Department of Internal Medicine, Stanford University School of Medicine, Stanford, CA

^3^ Department of Medicine, Saint George’s Hospital, University of London, London, UK

^4^ Division of Gastroenterology and Hepatology, University of Tennessee Health Science Center, Memphis, TN

^5^ Department of Research, United Network for Organ Sharing, Richmond, VA

**Corresponding Authors:**

Aijaz Ahmed, M.D., George Cholankeril, M.D.

750 Welch Road, # 210

Stanford University

Palo Alto, CA 94304, USA

Phone: 650-498-5691

Fax: 650-498-5692

E-mail: aijazahmed@stanford.edu

**Supplementary Table. Cumulative incidence post-transplant mortality rates among D-MELD cohorts.**

|  | **D-MELD 0-4**  **% (95% CI)** | **D-MELD 5-10**  **% (95% CI)** | **D-MELD >10**  **% (95% CI)** | ***P* value** |
| --- | --- | --- | --- | --- |
| **7-Day Mortality** | 1.61 (1.27-1.91) | 2.23 (1.72-2.88) | 2.29 (1.65-3.18) | 0.0413 |
| **30 Day Mortality** | 2.93 (2.59-3.31) | 3.73 (3.06-4.55) | 4.51 (3.57-5.68) | 0.0024 |
| **1-Year Mortality** | 9.91 (9.26-10.60) | 12.84 (11.54-14.28) | 13.63 (11.95-15.52) | <0.0001 |

Abbreviations: CI, confidence interval; D-MELD, delta change in Model for End-Stage Live Disease Score
